# Childcare workers’ experiences of supporting exclusive breastfeeding in Kuala Muda District, Malaysia: a qualitative study

**DOI:** 10.1186/s13006-016-0095-4

**Published:** 2017-01-06

**Authors:** Mohd Azri Mohd Suan, Azrina Ayob, Maheran Rodzali

**Affiliations:** 1 Clinical Research Centre, Sultanah Bahiyah Hospital, Alor Setar, Kedah Malaysia; 2 Sultan Abdul Halim Hospital, Sungai Petani, Kedah Malaysia; 3 Obstetrics and Gynaecology Department, Kepala Batas Hospital, Jalan Bertam 2, Kepala Batas, Penang Malaysia

**Keywords:** Child care, Breast feeding support, Breast feeding, exclusive, Qualitative research

## Abstract

**Background:**

The role of childcare workers at registered nurseries in supporting exclusive breastfeeding practice is important, as many newborn babies are placed in nurseries during working hours. To increase exclusive breastfeeding rates among working mothers, understanding childcare workers’ experiences and needs relating to supporting these mothers is crucial. This study aimed to explore childcare workers’ experiences of supporting breastfeeding at registered nurseries.

**Methods:**

We used a qualitative design to conduct in-depth, semi-structured interviews with ten childcare workers at seven registered nursery centres in Kuala Muda District, Malaysia. Attitudes towards exclusive breastfeeding practice, experiences of breastfeeding training and information, and experiences supporting exclusive breastfeeding at the nursery were explored. Participants were asked to suggest improvements for exclusive breastfeeding practice at their nursery. All interviews were audio recorded, transcribed verbatim, and translated before analysis.

**Results:**

All participants demonstrated a positive attitude in supporting and promoting exclusive breastfeeding practice, mainly centred on the advantages of breastfeeding. Various supports have been found such as labelling bottled breastmilk, allowing the mother to come to the nursery during breaks, and providing reading materials. However, several issues emerged that include parents’ choice on infant feeding practice, insufficient content on breastfeeding topics during training, and adherence to the (not recommended) practice of bottle feeding expressed breastmilk. Recommendations to enhance breastfeeding were also suggested by participants.

**Conclusion:**

Childcare workers may serve as another potential resource for sustaining exclusive breastfeeding at registered nurseries.

**Electronic supplementary material:**

The online version of this article (doi:10.1186/s13006-016-0095-4) contains supplementary material, which is available to authorized users.

## Background

Exclusive breastfeeding is the practice of giving a newborn baby only breastmilk (without supplementary nutrition) for the first six months of life. It is recommended that supplementary breastmilk then be provided after introduction of other foods until the infant is two years old [1, 2]. There is evidence on the advantages of breastmilk for infants and mothers compared with formula milk [2–5]. However, the rate of exclusive breastfeeding for the first six months remains low. Globally, only 37% of infants aged 0–6 months are exclusively breastfed, with the highest percentage (47%) among infants in the South-East Asia region [6, 7]. Locally, results from the Malaysia National Health and Morbidity Survey in 2006 showed that although 94.7% of women had ever breastfed their newborn babies, only 14.5% practised exclusive breastfeeding for the first six months [8].

The rate of successful exclusive breastfeeding is lower among working mothers. In a survey comprising 228,000 new mothers of varying employment status in the United States, the breastfeeding rate at six months post-delivery was statistically lower among mothers employed full time (26.1%), as compared with part-time (36.6%) and non-working mothers (35.0%) [9]. Similar disparities between employed and unemployed mothers were also seen in other studies [10, 11]. Returning to work, time costs, and unconducive workplaces were common reasons for early breastfeeding cessation among working mothers [12–14]. Similar to findings from these studies, working mothers in Peninsular Malaysia had a low rate (43.1%) of exclusive breastfeeding [15].

These statistics shows that it is essential to provide support to sustain exclusive breastfeeding practice especially among working mothers. Extensive studies have shown that intervention and support by medical professionals [16, 17], family members [18, 19], and peer support groups [20, 21], and use of media technology [22] have helped to boost breastfeeding rates. Employers’ support was also commonly mentioned by working mothers [23, 24]. Another support that may potentially influence breastfeeding practice but that is rarely referred to in the literature is that from childcare workers.

Many mothers need to leave their babies with a childcare worker or nursery to work. The longer the mother is at work, the longer the baby will spend time with the childcare worker. Hence, childcare workers and settings can be primary sources of support for working mothers who want to breastfeed by helping to store, handle and feed the baby with breastmilk while the mother is at work [25]. In studies examining childcare providers’ support of breastfeeding, Javanparast et al. [26] and Batan et al. [27] found that by helping to feed expressed breastmilk and allowing mothers to breastfeed before or after work, childcare providers helped mothers maintain breastfeeding at six months postpartum. Nevertheless, Lucas et al. [28] found some knowledge deficit among childcare providers in the area of health benefits and proper handling of breastmilk, indicating the need for adequate support and training for them to help working mothers.

With almost 5 million women employed in various sectors in Malaysia [29], many babies are cared for by childcare workers either at the childcare worker’s home or at a registered nursery while their mother is at work. Currently, formal training for childcare workers is conducted by a local government agency using the syllabus from the ‘Permata’ programme. This is a national education programme specifically designed to support the physical and intellectual development of children under four years old. It is compulsory for new childcare workers to attend this course, and nursery owners must attend as a prerequisite to renewing their nursery license.

Given the low rates of exclusive breastfeeding among working mothers and the opportunity for childcare workers to be involved in promoting and supporting exclusive breastfeeding, understanding the childcare workers’ experiences and needs at this time is crucial. Since such information is sparse in the local population, the aim of this study was to explore childcare workers’ experiences in supporting breastfeeding at registered nurseries in Kuala Muda District, in the state of Kedah, Malaysia.

## Methods

### Study design and setting

This exploratory qualitative research study, using face-to-face in-depth interviews was conducted with childcare workers at registered nursery centres in Kuala Muda District, Kedah, Malaysia. A list of all registered nurseries in the district was obtained from the Department of Social Welfare. This department, under the Ministry of Women, Family and Community Development, is responsible for licensing and monitoring the operation of all registered nurseries.

### Participants

Of the 43 registered nurseries in the list, ten had closed down, two had previously moved, and 15 nurseries did not cater for children younger than six months old. All childcare workers at the remaining 16 nurseries who had received formal training were invited to participate in the study via mail. Exclusion criteria were childcare workers who worked at the participating nurseries but did not take care of babies aged six months or younger and childcare workers with less than three months work experience. These exclusion criteria reflect the research focus (the current care of babies under six months). Information packs containing the invitation letter, a study information sheet, study approval information and authors/researchers contact numbers were posted to the 16 nurseries. The authors/researchers then contacted these nurseries and arranged an interview time with interested participants.

### Data collection and analysis

Following provision of written informed consent, in-depth face-to-face interviews were conducted with the childcare workers using a semi-structured interview guideline (Additional file 1). This guideline included open-ended questions about their experience in supporting exclusive breastfeeding in the nursery, experience with breastfeeding-related training, and suggestions to improve exclusive breastfeeding practice. Before the first interview session, a pilot interview was conducted with a pre-identified childcare worker to refine the questions and manner of questioning. Interviews were conducted at the respective nurseries, which also provided an opportunity to observe existing facilities.

Participants and researchers did not know each other, but participants were aware of the interviewers’ positions as medical researchers. Although the participants had read the study information sheet mailed earlier to them, the authors/researchers re-explained the purpose of the study before the interview session took place. The participants were also informed that information obtained during the interview session for this study would be kept and handled in a confidential manner, and any expressed views did not affect their application to renew nursery licenses in future. Informed consent was obtained after the participant was satisfied with these explanations.

Face-to-face interviews lasting 40–60 min were conducted in the national language (Bahasa Malaysia). Interviews continued until data saturation was reached and the interviewee no longer provided new information. An assistant took field notes on non-verbal cues. Interviews were audio-recorded, transcribed verbatim, and translated into English by two of the authors (MAMS and AA). Data from the interviews and field notes were used for triangulation.

Interview files were coded by all authors using both pre-determined and inductive categories. Codes were derived from the literature reviews and participant-generated constructs. All authors had regular discussion throughout the data analysis process. All data were handled and kept by the first author (MAMS).

### Ethics approval

This study was approved by the Medical Research Ethics Committee, Ministry of Health Malaysia (National Medical Research Register [NMRR]-14-259-20182). In addition, prior to undertaking the study, the study methods and purpose were presented to the representatives of the District Department of Social Welfare and their approval also obtained.

## Results

Ten childcare workers from seven registered nurseries consented to share their experiences of supporting exclusive breastfeeding at their respective nurseries. All were female with more than one year of experience in childcare. All babies cared for by these childcare workers were aged three months old and above. In all of the nurseries in which the participating childcare workers were employed there were at least two babies who were still being exclusively breastfed. There is no formal written policy on breastfeeding currently available at their respective nurseries. The characteristics of the ten participants are summarised in Table 1. Four key themes emerged from the data analysis: attitudes towards exclusive breastfeeding, the experience of breastfeeding training and information, experience supporting exclusive breastfeeding at nursery, and ideas to enhance breastfeeding practice at the nursery.

**Table 1:** Characteristics of interviewed childcare workers (*n* = 10)

ID	Position of Interviewee	Age of Interviewee (years)	Experience in childcare (years)	Highest level of education attained
#1	Childcare worker and nursery owner	38	10	Secondary school
#2	Childcare worker	27	1	Secondary school
#3	Childcare worker and nursery supervisor	43	12	Diploma
#4	Childcare worker and nursery owner	41	11	Secondary school
#5	Childcare worker	25	2	Secondary school
#6	Childcare worker and nursery owner	39	5	Secondary school
#7	Childcare worker and nursery owner	43	7	Secondary school
#8	Childcare worker	22	3	Secondary school
#9	Childcare worker and nursery owner	53	17	Secondary school
#10	Childcare worker and nursery owner	48	15	Diploma

### Attitudes towards exclusive breastfeeding

In general, all participants were supportive of exclusive breastfeeding. This positive attitude mainly centred on the advantages of exclusive breastfeeding. Most the participants were aware of the benefits of exclusive breastfeeding to the baby and were eager to support mothers who are willing to continue breastfeeding their babies when they return to work. Two participants shared their experiences on the advantages of breastmilk over formula milk on the babies’ health that motivated them to support exclusive breastfeeding at the nursery (comments throughout are paraphrased from the translations).

*“I have been working here for quite some time. I can see that babies receiving breastmilk are healthier than those on formula milk. That’s why I support exclusive breastfeeding.”*

*“Babies receiving breastfeeding are different. They are smart, and seldom have a fever or fall sick.”*

Ease of preparation for feeding was another aspect of bottled breastmilk preferred by the childcare workers.

*“Bottled breastmilk is much easier [to prepare]. Compared to formula milk, we only need to bring out the bottled breastmilk from the fridge, soak the bottle in warm water and can then start to feed the baby.”*

One of the participants also felt that exclusive breastfeeding helps to save parents money.

*“It’s quite expensive now to buy formula milk. I feel that mothers should breastfeed their baby at this age [0–6 months old] to save money.”*

This positive attitude was also shown by another participant, also an owner of the nursery, who made it compulsory for the parents of babies aged six months and younger to bring expressed breastmilk to the nursery.

*“I strongly advise the mothers to breastfeed their baby until 6 months old. That is my policy here [at the nursery]. I tell the mothers to express, store the breastmilk in the bottle and bring it here.”*

Although all participants supported exclusive breastfeeding, most of them felt that it was the personal choice of the mother or parents whether to continue breastfeeding. As a childcare worker, they believed their duty was to feed the babies with the milk (either breastmilk or formula milk) provided by the mothers.

*“We just follow the parents’ [choice of giving breastmilk or formula milk]. If they have supplied us with breastmilk, then we will feed their baby with breastmilk. If [provided with] formula milk, then we just feed them with that.”*

*“Most of the babies sent here at the age of three months old or older were already on mixed feeding [breastmilk and formula milk]. Their mother did not supply us with the bottled breastmilk, and so we can’t do anything about it.”*

### Experience of breastfeeding training and information

Participants were asked about the sources of information and any training received related to breastfeeding. Their experiences on these were categorised into sub-themes: (1) formal training and (2) other sources of education and information.

#### Formal training

All participants reported that they became aware of exclusive breastfeeding after attending courses conducted by a local government agency.

*“I received my training by attending the ‘Permata’ course.”*

*“I send all my new workers to this course by ‘Permata’. It is a good exposure for them as they don’t have any experience [in handling babies and children] before this.”*

However, several participants expressed their concern about the content of the breastfeeding topic during the course, which they claimed was too brief. Surprisingly, this course content was intentionally brief as the childcare workers were not mothers of newborn babies themselves.

*“The speaker [at the course] did not talk in detail about breastfeeding. They only mentioned it once, and that’s it. It was only brief because we are not the mothers of the babies. There was no practical session included during the course.”*

#### Other sources of education and information

Some participants shared their experience of gaining additional knowledge about exclusive breastfeeding from the Internet and even from the mother herself.

*“I also learned from the Internet how to prepare the breastmilk before giving it to the baby.”*

*“One of the mothers who enrolled her baby here is a doctor. She taught me how to prepare the breastmilk and how to label the bottle according to date.”*

One participant put several articles about breastfeeding up on the nursery wall for other childcare workers and mothers to read.

*“I put up some articles on babies’ nutrition and breastfeeding on the wall. We photocopied them from a magazine. I believe that many of my friends [other childcare workers] and even the parents don’t have time to read all of these at home.”*

### Experience supporting exclusive breastfeeding at nursery

All participants provided mothers with good support for exclusive breastfeeding practice, consistent with the knowledge gained from attending relevant courses. Two further sub-themes emerged from their experiences during supporting exclusive breastfeeding: (1) pleasant experiences and (2) distressing experiences.

#### Pleasant experiences

All participants helped to keep the bottled breastmilk refrigerated. Some also helped to label the bottles with the child’s name and date, if not already done by the mother.

*“We help to label [with name and date] the bottle. The mother just brings the bottle, and we will help them.”*

*“I will help to label the bottled breastmilk. So it would not be mistaken [for other babies’ milk] when we kept it in the fridge.”*

Furthermore, several participants encouraged the working mothers to send the pumped breastmilk or breastfeed their babies during lunch time/breaks.

*“For those mothers who work nearby, if they want to come here to breastfeed their baby, we allow them to do so. She can breastfeed for about 20 to 30 min. Then she returns to work.”*

*“I encouraged the mother to come over here during lunch break either to bring the [pumped] breastmilk or to breastfeed the baby.”*

#### Distressing experiences

Nevertheless, some childcare workers experienced difficulties in supporting and promoting exclusive breastfeeding. Difficulty in convincing the mother to sustain exclusive breastfeeding was the prominent issue voiced by many participants. Despite many babies still being exclusively breastfed, several working mothers had already started mixed feeding when they first enrolled their babies at the nursery at three months old. These mothers did not seem to respond favourably when advised to prolong the exclusive breastfeeding period.

*“Several parents provided us with both breastmilk and formula milk when their baby was first enrolled here. As a care provider, I have no choice except to feed the baby with any milk supplied to me.”*

*“Some of the babies sent here at 2–3 months old were already started on formula milk. If we talk about the advantages of giving breastmilk, the parents seem to not want to listen to us.”*

Another issue brought up by several participants was difficulty in feeding the baby with bottled breastmilk. Surprisingly, several participants placed the blame on the mothers for not training their babies with bottle feeding before returning to work.

*“The mother never tried bottle feeding at home. This makes it difficult for us [childcare workers] at the nursery to introduce it.”*

*“It was a tough time to start them [the babies] with bottle feeding at the nursery. The babies kept crying, and I had to try various methods such as bottle feeding, using a spoon and even a syringe. I needed to be patient to handle this situation.”*

### Ideas to enhance exclusive breastfeeding practice

The participants were asked to share their thoughts on how to improve breastfeeding support and create a more breastfeeding-friendly environment at their nursery. All agreed that information on exclusive breastfeeding practice should be regularly delivered to the childcare workers and the parents.

*“I agreed that these two parties [childcare workers and mothers] need to be informed more on exclusive breastfeeding on a regular basis.”*

To achieve this, most participants suggested a short talk on breastfeeding at the nursery given by a health care worker.

*“The health officer can come and give a talk about breastfeeding. I suggested such a talk could be done over the weekend, as the mothers have more free time to attend it. If the talk was given to us (childcare workers), we could learn something too, but it is much better if the talk is delivered to the parents.”*

One participant also suggested that exclusive breastfeeding practice could be boosted by a special certificate given to the nurseries with a childcare worker who is well trained on breastfeeding.

*“I would like to propose … those nurseries that have sent their staff to attend special training on breastfeeding are awarded with some sort of ‘breastfeeding-friendly nursery’ certificate. I can use this [certificate] to promote my nursery. And for mothers who are willing to continue to breastfeed their baby, they can look for such a nursery to enrol their baby when they return to work.”*

## Discussion

The rate of exclusive breastfeeding among working mothers worldwide is lower than the World Health Organisation (WHO) targets [9–11], with Malaysia being no exception [8, 15]. Many factors to boost breastfeeding rates have been documented. This study examined the role of childcare workers as another potential resource for sustaining exclusive breastfeeding by exploring their experiences of supporting breastfeeding at registered nurseries. Our data highlighted a positive attitude among participants toward supporting and promoting exclusive breastfeeding practice. Various methods of support were revealed through the interviews with childcare workers in our study, including helping to label bottled breastmilk, encouraging mothers to come over during breaks to breastfeed, and providing reading materials regarding breastfeeding to the mothers.

Our findings were consistent with other studies outcomes in the literature [27, 28, 30]. Batan et al. [27] also found that good support from childcare providers at three months may help mothers to prolong breastfeeding up to six months. This finding indicates the important role that can be played by childcare workers to enhance breastfeeding practice in the local population, as most of the babies were sent to them at the age of three months old, when most mothers return to work after their maternity leave is over.

Nevertheless, several issues raised by the participants need addressing. Concern regarding the decision to continue breastfeeding was the commonest problem expressed by the participants. They are obliged to follow any infant feeding practice (either exclusive breastfeeding or mixed feeding) determined by the parents. Furthermore, many participants felt that they were not in a position to advise the mother to continue and sustain exclusive breastfeeding. A similar problem was found by Javanparast et al. [26] and Cameron et al. [25] among childcare workers in Adelaide, Australia and North Carolina, who were supporting parental choice in infant feeding rather than playing their active role in breastfeeding promotion to the parents. In line with the suggestion from the previous studies, childcare workers need to be empowered to promote breastfeeding at their nursery. Since there is no standard policy on breastfeeding available currently at nurseries, having a formal written policy may assist the childcare workers with the decision-making process and help them to understand better their role in breastfeeding support.

Additionally, all participants in our study mentioned their participation in the Permata breastfeeding course, a government programme for workers in early childhood education. This programme reflects the strong government commitment in Malaysia to enhance breastfeeding practice at nurseries. However, the participants’ feedback on the brevity of content on breastfeeding topics, and the lack of practical sessions, should be considered by the course organiser. Participants’ positions as childcare workers should not be used as a reason to provide only a brief talk on breastfeeding, as childcare workers have an important role to play in promoting exclusive breastfeeding. It is also necessary to ensure that childcare workers have comprehensive, up to date knowledge about how to correctly store, label, and prepare breastmilk. The training course is essential and should cover a broad range of breastfeeding topics such as basic breastfeeding management, counselling techniques and nutrition [31]. Consistent with the theme of international initiatives such as World Breastfeeding Week 2016 [32], information on the new Sustainable Development Goals and ways to value breastfeeding as central to achieving these goals could also be addressed during the training course. In addition to conventional methods, the course organiser could include training techniques such as demonstrations using a mannequin, videos, and group work exercises.

Our study also highlighted that the practice of bottle feeding with expressed breastmilk was common in local nurseries. This practice is not consistent with the International Code of Marketing of Breastmilk Substitutes by the WHO [33], which discourages the use of bottles to feed babies. In addition to the gaps in the training course content as mentioned above, this inappropriate practice suggests there is a lack of essential messages about breastfeeding conveyed to childcare workers during the course. Local stakeholders and policy makers should be engaged in reviewing and strengthening the training syllabus for childcare workers. Dissemination of appropriate breastfeeding information (including labelling, storing, and preparing breastmilk) should be prioritised to better inform and educate childcare workers. Furthermore, feedback from participants in the present study reflected their preference for using a bottle rather than a cup or spoon to feed expressed breastmilk to babies. The reason for this preference is unknown and is an important area to explore in a future study.

Improving awareness of breastfeeding practice both for the mothers and childcare workers was a prominent suggestion by participants to enhance exclusive breastfeeding practice. As suggested by one participant who was also a nursery owner, talks delivered by healthcare staff to the parents at the nursery on the importance of sustaining exclusive breastfeeding may be beneficial. Mothers who have managed to breastfeed exclusively could also be called on to share their successful experience in providing breastmilk to their babies. To reach a wider audience, this type of awareness dialogue and sharing of experiences could be conducted at a community level during World Breastfeeding Week. Evidence shows that engaging with friends and family members who had previously breastfed a child had a positive influence on the working mother to continue breastfeeding during employment [20, 21]. Another suggestion was to acknowledge nurseries with staff/childcare workers well trained on breastfeeding management and counselling. In keeping with the idea mentioned by one participant, such an initiative may benefit both the nursery and parents who intend to breastfeed exclusively.

Although this study analysis was based on responses from childcare workers themselves, several limitations should be noted. The participants were sampled from nurseries registered with the government authorities. An appropriate next step would be to involve childcare workers working at home. Given the fact that such childcare workers are not required to register with local authorities and thus not to attend the formal course, their attitudes and experiences toward breastfeeding may differ. Second, the qualitative design means we were unable to generalise the findings. Despite its limitations, this study obtained valuable views and revealed several issues hindering exclusive breastfeeding practice at registered nurseries in the local population. The results may be useful for local authorities in future policy implementation, especially for targeted community breastfeeding programmes and resource/financial allocation. Several recommendations to enhance breastfeeding practice have been noted, and their implementation is necessary to increase the exclusive breastfeeding rates among working mothers.

## Conclusions

Practising exclusive breastfeeding after returning to work requires various forms of support to the mother. Childcare workers seem to provide useful assistance to working mothers based on their positive attitudes and experience. To encourage a more active role of childcare workers in promoting exclusive breastfeeding at their respective nurseries, various empowerment activities including implementation of standard policies and related training courses need to be provided for them. Childcare workers also require aid from the mother to ease the breastfeeding transition process into the nursery’s care. The availability of supports from childcare workers will facilitate exclusive breastfeeding and prolong its duration.

## Additional file

Additional file 1: Interview Guideline. (DOC 26 kb)

## Abbreviations

NMRR: National Medical Research Register
WHO: World Health Organisation
