# Supplementary material for: Childcare workers’ experiences of supporting exclusive breastfeeding in Kuala Muda District, Malaysia: a qualitative study
Source: Int Breastfeed J. 2017 Jan 6;12:2. doi: 10.1186/s13006-016-0095-4 (PMC5217309; doi:10.1186/s13006-016-0095-4)
Supplement: Additional file 1: — Interview Guideline. (DOC 26 kb) [file 13006_2016_95_MOESM1_ESM.doc]

**Additional file 1: Interview Guideline**

**Objective**

To explore childcare workers’ experiences of supporting breastfeeding at registered nurseries.

**Participants’ characteristics data**

1. May I know your position in this nursery?
2. May I know your age and highest education attained?
3. How long you have been involved in childcare?
4. How long this nursery has been operating?
5. How many babies aged 6 months old or under are in this nursery?

**Experiences of supporting breastfeeding practice**

1. What do you know about exclusive breastfeeding?
2. What support do you provide for exclusive breastfeeding? (prompt if the following issues are not raised spontaneously during the interview session);

- Support for the mothers
- Support on practice implementation
- Others

1. In your experience, is there any problem dealing with exclusive breastfeeding practice?
2. Can you tell me about any training or sources of information you have received on exclusive breastfeeding? (explore both formal and informal training / education / information received)
3. In your opinion, what would be effective ways to improve the practice of exclusive breastfeeding?

**Ending**

Thank you so much for your time so far.

That completes the questions.

Before we end this session, do you have anything else to add?

Thanks again for your willingness to answer these questions.
